# Case report: ranavirus infections in captive eastern box turtles (*Terrapene carolina carolina*) in Japan

**DOI:** 10.3389/fvets.2025.1627913

**Published:** 2025-08-29

**Authors:** Kenichi Tamukai, Hiroshi Shimoda, Sho Kadekaru, Yumi Une

**Affiliations:** ^1^Den-en-chofu Animal Hospital, Tokyo, Japan; ^2^Laboratory of Veterinary Microbiology, Joint Faculty of Veterinary Medicine, Yamaguchi University, Yamaguchi, Japan; ^3^Laboratory of Veterinary Pathology, Okayama University of Science, Okayama, Japan; ^4^Biomedical Science Examination and Research Center, Okayama University of Science, Okayama, Japan; ^5^The Animal Disease Research and Support Association, Tokyo, Japan

**Keywords:** box turtle, frog virus 3, Iridoviridae, ranavirus, reptile

## Abstract

Ranaviruses are broad host-range pathogens that cause fatal infections in ectothermic vertebrates, including fish, amphibians, and reptiles and are considered emerging infectious diseases. This report describes a case of ranavirus infection identified at a box turtle breeding facility in Japan. Of 12 box turtles, representing three species and housed in a mixed-species outdoor pen, all six eastern box turtles (*Terrapene carolina carolina*) exhibited clinical signs, and three died. Severe clinical signs in four turtles included nasal and oral discharge and palpebral edema, while two showed only oral discharge. No other species were affected. The oral and tongue mucosa were enlarged or partially eroded, with their surfaces covered by a pseudomembranous crust containing fibrin, heterophils, necrotic material, and multifocal bacterial colonies. Multifocal necrosis was observed in both the spleen and liver; however, no inclusions were detected in any affected tissues. Electron microscopy revealed cytoplasmic ranavirus-like particles within necrotic spleen cells. Ranavirus infection was confirmed by PCR, and partial genome sequencing identified a strain similar to frog virus 3 (FV3).

## 1 Introduction

Ranaviruses, members of the family Iridoviridae, are large (150–170 nm), icosahedral, double-stranded DNA viruses that infect ectothermic vertebrates such as fish, amphibians, and reptiles. These viruses can cause infection and disease across a wide range of species and represent a significant threat to amphibian and reptile populations worldwide (1–3). Ranavirus infections have been reported in chelonians, lizards, and snakes, most often being described in chelonians (4–6). Three recognized ranavirus species that infect reptiles include frog virus 3 (FV3), common midwife toad virus (CMTV), and epizootic hematopoietic necrosis virus (EHNV) (4–7). FV3 belongs to the species *Ranavirus rana1*, which also includes tiger frog virus (TFV), Bohle iridovirus (BIV), soft-shelled turtle virus (STIV), and other viruses. It is considered the most widely distributed ranavirus worldwide (7, 8).

North American box turtles belong to the genus *Terrapene*, which includes seven recognized species. The eastern box turtle (*Terrapene carolina carolina*), a subspecies of the common box turtle (*Terrapene carolina*), is distributed throughout the eastern United States, from Maine in the north to Texas in the west. This turtle faces threats from habitat destruction and fragmentation (9). In addition, ranaviruses have been identified as a contributing factor to population declines in this species (10, 11). North American box turtles are popular pets in Japan. Only a small number of captive-bred box turtles, legally obtained for the pet trade in the United States, are imported into Japan. Additionally, box turtles are bred, kept in captivity, and distributed domestically.

Ranaviruses have been detected in 24 turtle species from eight countries, in both captive and wild populations (12–15). The eastern box turtle has the highest number of documented ranavirus infections among reptile species (11, 16–21), with all reported cases originating from the United States. In Asia, only three cases of ranavirus infection in turtles have been reported: in farmed soft-shelled turtles [*Pelodiscus (Trionyx) sinensis*], an aquarium-held alligator snapping turtle (*Macrochelys temminckii*), and a yellow pond turtle (*Mauremys mutica*) from pet markets, all in China (15, 22, 23). In Japan, ranaviruses have been implicated in outbreaks involving wild American bullfrogs (*Lithobates catesbeianus*), captive poison dart frogs (*Dendrobates* and *Phyllobates* spp.), salamanders (*Hynobius nebulosus*), and captive lizards (*Pogona vitticeps*) (24–27). This report describes the first documented outbreak of ranavirus infection in turtles in Japan.

## 2 Case

### 2.1 Case description

Twelve adult female box turtles had been individually housed indoors for over 7 years in Tokyo, Japan, by a private breeder. The collection consisted of six eastern box turtles (*Terrapene carolina carolina*), three Florida box turtles (*Terrapene carolina bauri*), and three Chinese box turtles (*Cuora flavomarginata*). The turtles were fed a commercial omnivorous reptile pellet, Mynah bird food, various fruits, a small amount of meat, and crickets. During this period, all turtles remained clinically healthy.

In August 2022, they were moved to an outdoor pen (4 square meters) in the countryside (Chiba Prefecture, Japan) for environmental enrichment. The pen contained natural flora and a constant puddle of water drawn from a well. The area surrounding the pen was inhabited by sympatric amphibian species, including the Japanese brown frog (*Rana japonica*), Japanese tree frog (*Hyla japonica*), Schlegel’s Green Tree Frog (*Zhangixalus schlegelii*), and American bullfrog (*Lithobates catesbeianus*).

**Figure 1:**
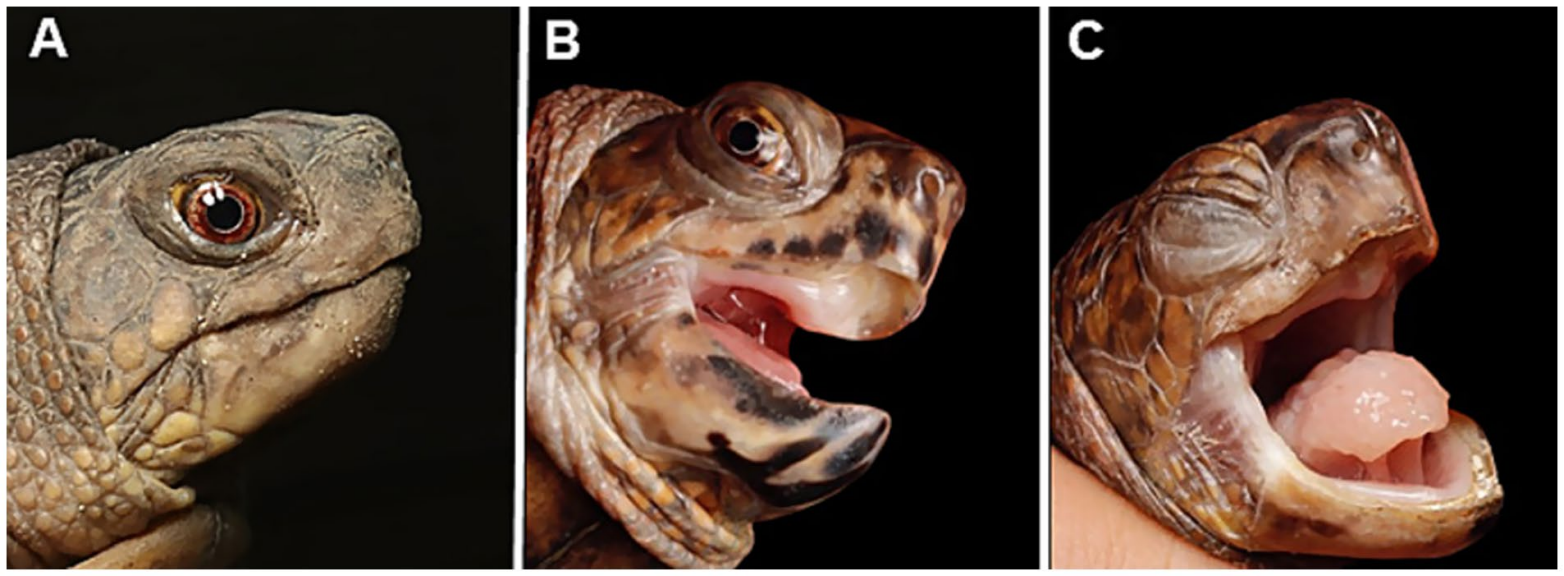
Appearance of captive eastern box turtles (*Terrapene carolina carolina*) involved in a ranavirus outbreak in Japan. **(A)** A clinically healthy individual. **(B)** Mild case showing open-mouth breathing and oral discharge. **(C)** Severe case exhibiting palpebral edema and closed eyes. The tongue is pale, suggesting anemia, and swollen, indicating glossitis.

In October 2022, all eastern box turtles exhibited clinical signs. Four turtles were severely affected, presenting nasal and oral discharge and facial edema, while two turtles had mild signs, showing only increased oral discharge (Figure 1). One turtle died 6 days after the initial onset of clinical signs, and two others succumbed 10 days post-onset. No clinical signs were observed in the other box turtle species. The surviving three eastern box turtles were isolated and treated with clindamycin (20 mg/kg, orally) and Lugol’s solution to disinfect the oral cavity.

### 2.2 Pathological examination

Pathological examinations were performed on two of the three eastern box turtles that died. The remaining individual was severely autolyzed, so no pathological sampling was attempted. Tissue samples (lung, trachea, bronchi, heart, liver, stomach, intestine, spleen, and kidneys) were fixed in 10% neutral-buffered formalin, processed routinely, and stained with hematoxylin and eosin (H&E) for histologic examination. For electron microscopic examination, the formalin-fixed lung was washed in 0.1 M phosphate buffer solution and post-fixed in osmium tetroxide. The fixed specimens were dehydrated in alcohol and embedded in epoxy resin using standard techniques. Ultrathin sections were stained with uranyl acetate and lead citrate and examined with a transmission electron microscope (JEM-1400Flash; JEOL, Tokyo, Japan).

**Figure 2:**
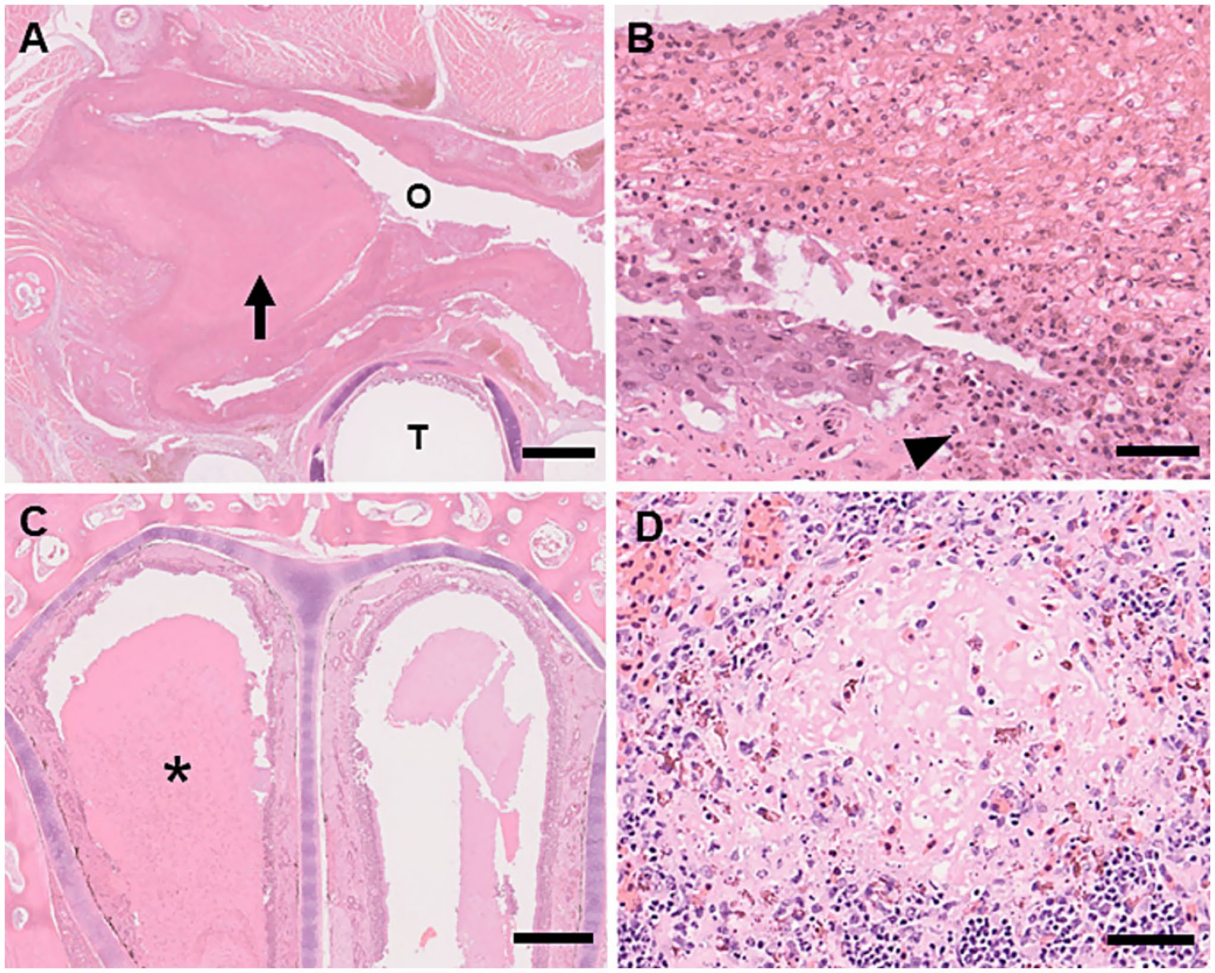
Histologic lesions of ranavirus infection in captive eastern box turtles (*Terrapene carolina carolina*) in Japan. H&E stain. **(A)** Necrotizing stomatitis. The epithelial surface of the oral mucosa is thickened due to a prominent pseudomembranous crust (arrow) Oral cavity (O). Trachea (T). Scale bar represents 1 mm. **(B)** High magnification of necrotizing stomatitis. The oral surface is covered by pseudomembranous crust consisting of fibrin, heterophils, and necrotic debris. Necrosis of the mucosal epithelium is observed (arrowhead). Scale bar represents 50 μm. **(C)** Necrotizing rhinitis. The nasal cavity is filled with serous discharge and necrotic debris (asterisk). H&E stain. Scale bar represents 500 μm. **(D)** Multifocal necrosis in the spleen: Focal necrosis with associated heterophilic infiltration. H&E stain. Scale bar represents 50 μm.

**Figure 3:**
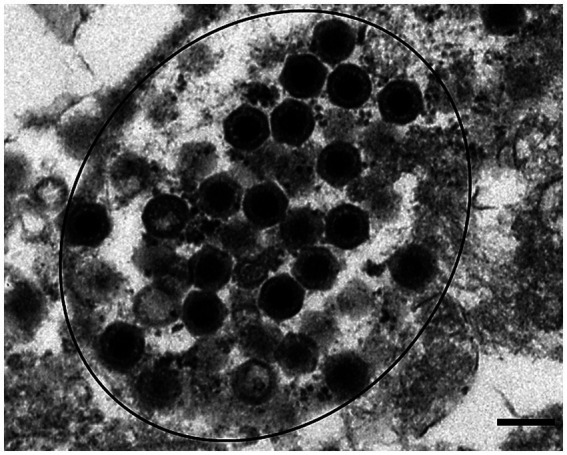
Icosahedral virus particles consistent with ranavirus were observed in spleen tissues (circled black). Transmission electron micrograph. Scale bar represents 200 nm.

A common histopathological feature in multiple organs was necrotic lesions. The oral, esophageal, and nasal cavities contained necrotic inflammatory materials. The oral and tongue mucosa were largely or partially missing, and their surfaces were covered by pseudomembranous crusts containing fibrin, heterophils, necrotic debris, and multifocal bacterial colonies. Multifocal necrosis, including heterophil infiltration, was observed in the spleen and liver (Figure 2). Electron microscopy revealed ranavirus-like particles within degenerative cells in the spleen. These particles were icosahedral in shape and approximately 150 nm in diameter (Figure 3).

### 2.3 Detection of viral genes, sequence, and phylogenetic analysis

Kidney, spleen, liver tissues, and oral swabs were collected from the deceased individuals. One month after the last eastern box turtle death, oral swabs were collected from all remaining turtles in the pen, including three eastern box turtles, three Florida box turtles, and three Chinese box turtles, for molecular analysis. All of the turtles appeared clinically healthy at the time of sampling. All samples were homogenized or suspended in phosphate-buffered saline (PBS). The supernatant or suspended samples were used for viral gene detection. DNA was extracted using the DNeasy Blood & Tissue Kit (QIAGEN, Hilden, Germany) according to the manufacturer’s instructions.

For the detection of mycoplasma, herpesvirus, and ranavirus, the DNA samples were subjected to PCR using the TaKaRa Ex Taq kit (Takara Bio, Otsu, Japan). Universal mycoplasma primers (GPO3F/MGSO) targeting the 16S rRNA gene were used for pan-mycoplasma detection (28). The PCR reaction followed a multistep program consisting of an initial denaturation at 96 °C for 2 min, 35 cycles of denaturation at 96 °C for 20 s, annealing at 55 °C for 30 s, and extension at 72 °C for 30 s, and final extension at 72 °C for 5 min. Pan-herpesvirus primers were used to detect herpesvirus DNA polymerase via nested PCR, as described previously (29). The first and second rounds of the reactions each followed a multistep program consisting of an initial denaturation at 94 °C for 5 min, 45 cycles of denaturation at 94 °C for 30 s, annealing at 46 °C for 30 s, and extension at 72 °C for 1 min; and a final extension at 72 °C for 5 min. Pan-ranavirus primers were used to detect the ranavirus DNA polymerase, as previously described (30). The PCR program included an initial denaturation at 94 °C for 2 min, 35 cycles of denaturation at 94 °C for 30 s, annealing at 55 °C for 30 s, and extension at 72 °C for 1 min, followed by a final extension at 72 °C for 1 min. All primers used in this study are listed in Supplementary Table S1. Ranavirus DNA polymerase genes were detected in the eastern box turtles, including three deceased individuals and one that had recovered from a moribund state. No mycoplasma or herpesvirus DNA was detected in any sample. Additionally, all other turtle species tested negative for ranavirus, herpesvirus, and mycoplasma.

**Figure 4:**
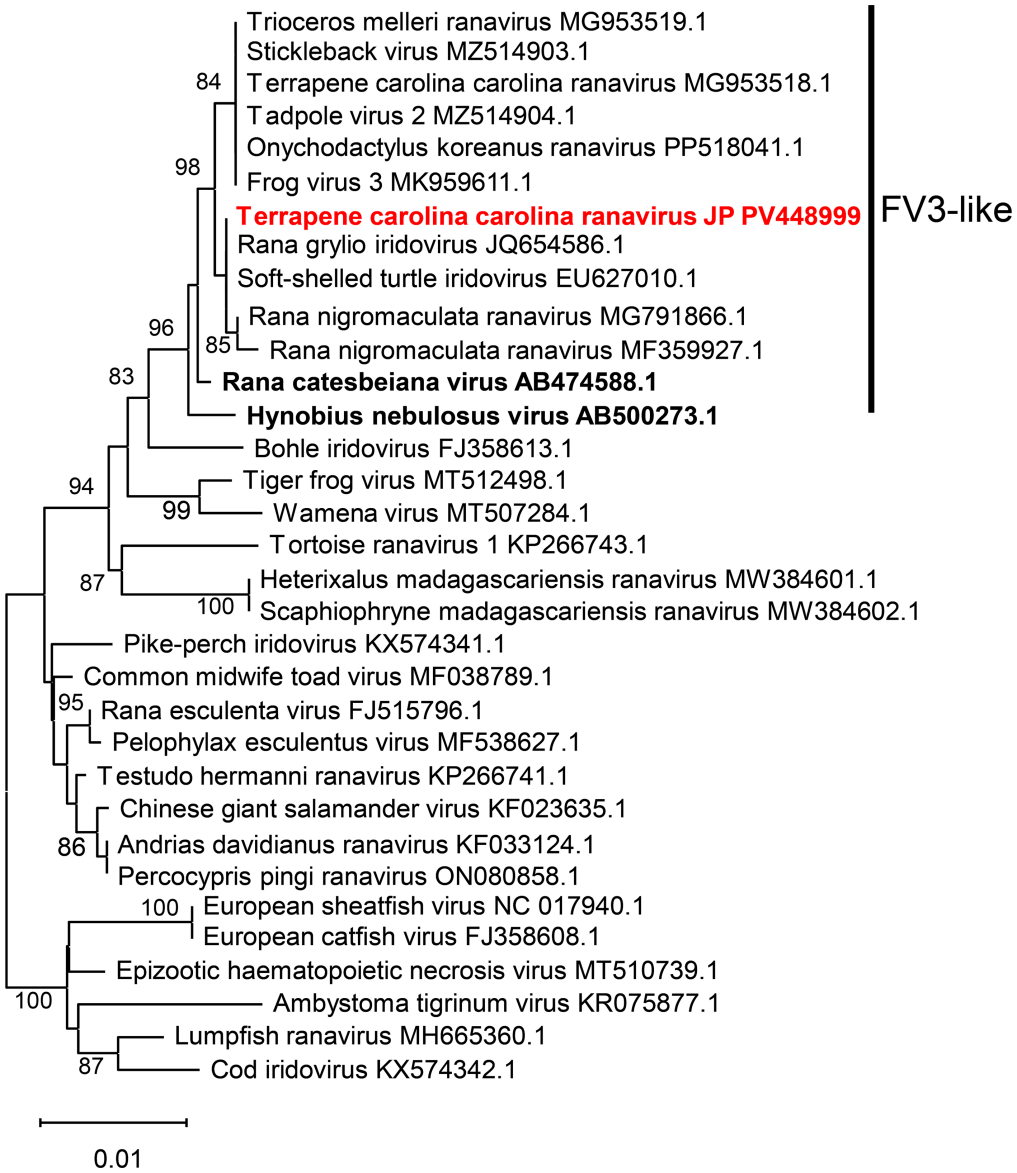
Phylogenetic tree of the detected ranaviruses based on the full amino acid sequences of the major capsid protein. This phylogenetic tree was constructed using 464 amino acids from the MCP gene. Sequences of the ranavirus detected in this study are shown in red. Sequences of ranaviruses previously identified in amphibians in Japan are shown in bold. GenBank accession numbers are provided for the listed viruses.

To perform a phylogenetic analysis of the detected ranavirus, the full sequence of the major capsid protein (MCP) gene was determined from a ranavirus-positive kidney sample of a deceased box turtle. The reaction was conducted using FV 3-specific primers listed in Supplementary Table S1, following the program used for pan-ranavirus PCR. Amplified fragments were purified using either the MinElute® PCR Purification Kit (QIAGEN) or the MinElute Gel Extraction Kit (QIAGEN), according to the manufacturer’s instructions. Purified fragments were submitted to Eurofins Genomics (Tokyo, Japan) for nucleotide sequencing using the Sanger method. Phylogenetic analysis was performed using the Molecular Evolutionary Genetics Analysis (MEGA) software package (version 11). After primer trimming, amino acid sequences predicted from the detected DNA sequences and reference ranavirus sequences from GenBank were aligned using ClustalW. Neighbor-joining trees were constructed in MEGA 11 using a p-distance model and 1,000 bootstrap replicates. The full MCP gene sequence determined in this study was deposited in GenBank (accession no. PV448999). The detected ranavirus has been provisionally named *Terrapene carolina carolina* ranavirus JP. Phylogenetic analysis placed the detected ranavirus within the FV3-like group (Figure 4). In addition, pairwise sequence alignment of the MCP gene revealed 100% nucleotide identity with *Rana grylio* iridovirus (JQ654586) and soft-shelled turtle iridovirus (EU627010), 99.71–100% identity with other FV3-like viruses and 99.67% identity with previously reported ranavirus identified in eastern box turtle (MG953518).

## 3 Discussion

Eastern box turtles infected with ranaviruses have been reported to exhibit clinical signs, including palpebral edema, rhinitis, and stomatitis (11, 18, 31, 32). These signs are often systemic and fatal, with the virus most highly concentrated in the kidney, spleen, and liver (31–33). In this case, histopathological lesions included varying degrees of necrosis and degeneration in the hepatocytes and spleen, with heterophil infiltration. Although intracytoplasmic inclusion bodies were not detected histologically in any tissue, ranavirus-like particles were observed in the spleen, and FV3-like genes were detected in the organs and oral swabs. Clinical signs in turtles infected with ranavirus are often similar to those caused by *Mycoplasma* spp. and herpesvirus infections (12), but no evidence of these infections was found in this case. The clinical signs and histological findings were consistent with previous reports on ranavirus in turtles (10, 11, 32), confirming this as an outbreak of ranavirus infections in eastern box turtles.

There are no known effective antivirals for ranaviral disease. However, early medical intervention, including supportive care and antibiotic treatment, is considered a key factor in the favorable outcome for captive eastern box turtles (34). In this case, heterophil infiltration was observed in internal organs, and bacterial infection was detected in the oral mucosa and esophagus. Ranaviral infections can be accompanied by secondary pathogens that may exacerbate the disease (18, 27, 33–35). The surviving turtles received antibiotic treatment and oral disinfectants, which may have helped prevent clinical deterioration due to secondary bacterial infection. However, it is possible that selecting antibiotics based on culture and sensitivity test, combined with supportive care such as fluid therapy and pain management, could have further improved treatment efficacy.

In a previous study, although clinical signs had resolved in free-living three-toed box turtles (*Terrapene mexicana triunguis*), FV3 was still cultured and detected by PCR 1 month later (33). In the present case, one of the three surviving eastern box turtles tested positive for ranavirus by PCR despite being asymptomatic and clinically healthy at the time of oral swab collection. This individual had previously exhibited severe clinical signs during the outbreak. These results support the possibility of an asymptomatic carrier state in turtles (3, 20, 31, 33, 35–37).

Ranavirus virulence is influenced by factors such as host species, host density, immune status, environmental conditions, and seasonality (38). In this study, other box turtle species housed in the same pen exhibited no clinical signs and tested negative for ranavirus by PCR. It remains unclear whether these negative results were due to differences in host susceptibility or an effective immune response that inhibited viral infection. These findings further confirm that the eastern box turtle is highly susceptible to ranavirus, including in outdoor environments in Japan. However, the relationship between host range and pathogenicity remains poorly understood. Further research is needed to better understand how these infections are affecting the health of different turtle species.

Ranavirus infection in chelonians may be linked to increased exposure to infected sympatric amphibians. This exposure may occur through predation on infected individuals or contact with water contaminated by viruses shed from amphibian hosts (3, 17). Additionally, studies have indicated a significantly higher probability of FV3 detection in box turtles residing in moist microhabitats co-inhabited by FV3-positive amphibians (10). According to the owner, American bullfrog juvenile frogs occasionally invaded the pen in October, and box turtles were observed eating the dead frogs. In Japan, mass die-offs of North American bullfrog larvae due to ranavirus infection have been reported from early September to mid-October (25). Ranavirus infections in chelonians are likely a consequence of spillover from amphibian mortality events (17). In an experimental infection study with ranavirus in turtles, mortality occurred 22–29 days post-inoculation (18, 31, 39). In our case, the outbreak was observed approximately 1 month after the bullfrogs appeared in the pen, which was consistent with the incubation period observed in the infection experiments.

The onset of ranavirus infection in turtles is influenced not only by the affected species and exposure dose but also by the developmental stage and environmental temperature (39, 40). Decreases in environmental temperature have been correlated with increased mortality in eastern box turtles (40). According to the Japan Meteorological Agency, the mean monthly temperatures in Chiba Prefecture from August to October 2022 were 26.7 °C, 23.7 °C, and 17.0 °C, respectively, representing a drop of approximately 10 °C over 3 months. In the present case, testing for prior infection before the outbreak following the movement of the animals was not performed; therefore, the possibility of inapparent infections could not be eliminated. Additionally, the presence of ranavirus in the amphibians and water sources within the pen area was not feasible. As a result, horizontal transmission from sympatric amphibians could not be confirmed due to lack of supporting evidence. This outbreak was likely facilitated by a combination of host susceptibility, animal movement, and declining environmental temperatures.

In conclusion, this report describes the first documented case of ranavirus infection in turtles in Japan. Additionally, molecular analysis, along with previous reports, provides evidence that several FV3-like viruses are circulating in the country (25, 26). Our case suggests that the pet trade of asymptomatic carriers may have played a role in the spread of ranaviruses. Japan is home to 11 species of freshwater turtles, including two endemic species and two endemic subspecies (41); however, no surveys of ranavirus infection have been conducted on these species. Further research is needed to understand the pathogenicity and epidemiology of ranavirus in Japan’s turtle populations.

## Data Availability

The datasets presented in this study can be found in online repositories. The names of the repository/repositories and accession number(s) can be found in the article/Supplementary material.
