# Supplementary material for: Case report: ranavirus infections in captive eastern box turtles (Terrapene carolina carolina) in Japan
Source: Front Vet Sci. 2025 Aug 29;12:1627913. doi: 10.3389/fvets.2025.1627913 (PMC12427586; doi:10.3389/fvets.2025.1627913)
Supplement: Supplementary file 1 [file Table_1.docx]

Supplementary Material

# Supplementary Table S1. List of primers used in this study for PCR analysis

| **Primer** | **Target gene** | **PCR** | **Sequence (5'-3')** | **Reference** |
| --- | --- | --- | --- | --- |
| GPO3 | Mycoplasma 16S rRNA | Single | GGGAGCAAACACGATAGATACCCT | 28 |
| MGSO |  | Single | TGCACCATCTGTCACTCTGTTAACCTC | 28 |
| DFA | Herpesvirus DNA Polymerase | 1st | AAGAATGGGAGGGGAAGAACC | 29 |
| ILK |  | 1st | TCCTGGACAAGCAGCARNYSGCNMTNAA | 29 |
| KGI |  | 2nd | GTCTTGCTCACCAGNTCNACNCCYTT | 29 |
| IYG |  | 2nd | CACAGAGTCCGTRTCNCCRTADAT | 29 |
| TGV |  | 2nd | TGTAACTCGGTGTAYGGNTTYACNGGNGT | 29 |
| Rana-DNApol-F | Ranavirus  DNA polymerase | Single | GTGTAYCAGTGGTTTTGCGAC | 30 |
| Rana-DNApol-R |  | Single | TCGTCTCCGGGYCTGTCTTT | 30 |
| MCP_1F | Ranavirus  Major Capsid protein | Single | ACTGTGTATCTTATAATAAA | This study |
| MCP_1R |  | Single | TTTGCATATCCCTCCAAAGAGAGG | This study |
| MCP_2F |  | Single | ATGGAGGACCCATGACGGAA | This study |
| MCP_2R |  | Single | ATGATAGGCAACACCAGCGA | This study |
| MCP_3F |  | Single | GCGCTCTTGACGGGATCTAC | This study |
| MCP_3R |  | Single | GGCTCCAATTACACCTGCGT | This study |
